# Silica Microspheres-in-Pores Composite Monoliths with Fluorescence and Potential for Water Remediation

**DOI:** 10.3390/nano11102681

**Published:** 2021-10-12

**Authors:** Adham Ahmed, Peter Myers, Haifei Zhang

**Affiliations:** 1 Department of Chemistry, University of Liverpool, Oxford Street, Liverpool L69 7ZD, UK; adham362@gmail.com (A.A.); Peter.Myers@liverpool.ac.uk (P.M.); 2 Thermo Fisher Scientific, Runcorn WA7 1TA, UK

**Keywords:** silica microspheres, emulsion templating, ship-in-a-bottle synthesis, silica–polymer composites, water remediation

## Abstract

Water pollution is a severe worldwide issue. Constructing advanced porous composite materials has been an efficient route to water remediation via adsorption. In this study, a unique microspheres-in-pores monolithic structure was fabricated. An emulsion-templated polymer monolith was first prepared and silica microspheres were subsequently formed in the porous polymer. A silica precursor was modified with a fluorescent dye and co-condensed with other precursors to fabricate porous composites with fluorescent properties, which were enhanced by the presence of Ag nanoparticles in the polymer matrix. This unique material showed good promise in water remediation by removing organic dyes and heavy metal ions from wastewater via a flowing filter or monolithic column separation.

## 1. Introduction

Hybrid or composite materials comprise more than one type of material and combine different properties, which can enhance their performance for various applications [1]. The incorporation of porosity and inducing high surface areas into hybrid materials are critical for some applications such as catalysis, separation, tissue engineering and energy storage [1,2,3]. There are various strategies to prepare hybrid materials. Commonly, different components or porous scaffolds/particulates with other components are blended, at the macroscopic level or microscopic level, followed by physical solidification or chemical reaction [1,2,3,4,5]. However, the main disadvantages are the difficulty in controlling the component morphology/property and minimising the gradual leaching of encapsulated particles.

The ship-in-a-bottle method is an effective approach to form larger guest molecules or particles in the pores with smaller windows [6,7,8,9,10]. The guest molecules and particles can exist as free-moving items, with a fully accessible surface via the small windows on the pore wall. At the same time, the guest molecules/particles are fully encapsulated and without the danger of being leached out. To prepare hybrid materials by the ship-in-a-bottle approach, precursors are usually diffused into the pre-fabricated pores through the windows on the pore wall. Larger guest molecules and more often nanoparticles are formed from the precursors in situ inside the pores. These particles are larger than the pore windows and thus encapsulated within the pores. Most reports by the ship-in-a-bottle approach have focused on preparing nanospheres or nanodots within mesopores or micropores [6,7,8,9,10]. Huang et al. reported the synthesis of intermetallic nanoparticles within mesoporous silica nanoparticles. The PtSn nanoparticles with a mesoporous silica shell showed high performance and close to 100% selectivity in furfural hydrogenation for furfuryl alcohol [6]. Amooghin and co-authors synthesised a Co–organic complex in zeolite Y nanoparticles which were further used to fabricate mixed-matrix membranes for the separation of CO_2_/CH_4_ with high permeability and high selectivity [7]. A metal–organic framework (MOF-808-P) was used as the microporous host for the confined synthesis of RuO_2_ nanoparticles. The resulting material exhibited high catalytic CO oxidation at low temperature (<150 °C) [8]. Polymer dots were formed inside hollow silica and carbon nanospheres. The polymer dots showed strong photoluminescence and could be used effectively to detect Cu^2+^. The fluorescence intensity decreased after adsorbing and chelating Cu^2+^ by the polymer dots within the porous shell [9]. Recently, carbon dots were fabricated within the zeolite SAPO (Silicoaluminophosphate)-20 particles by in situ pyrolysis of the organic entity trapped in the zeolite micropores during the synthesis. The carbon dots displayed a higher quantum yield of green fluorescence, compared with nonencapsulated carbon dots. This property was successfully explored for cellular imaging [10].

Although confined nanoparticles within micropores/mesopores can offer unique geometry, a protected surface and excellent catalytic/opto-electronic properties, the small mesopores and micropores can be disadvantageous for applications where mass transport is the bottle-neck step [2]. Macroporous materials are commonly fabricated by templating methods [11], with ice templating and emulsion templating (with high internal phase emulsions, HIPEs) being highly effective for the preparation of highly interconnected porous materials [12,13]. Hierarchically porous materials and hybrid materials, fabricated by employing multiple templates, are highly useful as adsorbents for water treatment, as scaffolds for controlled release and as supports for energy storage and catalysis [2,3,14]. Microspheres have a range of unique applications [15]. Hybrid materials by combining microspheres and macroporous materials can offer better properties for enhanced applications. However, there has been no or limited reports on the fabrication of hybrid materials with microspheres and macroporous materials via the ship-in-a-bottle approach. Such hybrid materials may give rise to highly useful properties as has been demonstrated in the nanospheres/nanopores systems [6,7,8,9,10].

Water pollution is a global issue which has resulted in grave consequences on humanity and the environment, particularly in developing countries [16,17]. Various advanced functional materials have been designed and developed for water remediation [18,19]. Herein, we reported the synthesis of silica microspheres within emulsion-templated polymer monoliths via the ship-in-a-bottle approach for wastewater treatment. By using (3-mercaptopropyl)trimethoxysilane and other silica precursors, silica microspheres with different functional groups (e.g., –SH, –NH_2_ and vinyl groups) could be formed and encapsulated in the emulsion-templated macroporous polyacrylamide. The porous composites were further modified showing fluorescence, enhanced by the presence of Ag nanoparticles in the polymer matrix. This provided a potential platform for detection of metal ions. A superior performance of removing dyes and metal ions from water using the hybrid materials was demonstrated.

## 2. Materials and Methods

### 2.1. Chemical and Reagents

Ammonium persulfate (APS, 98%), acrylamide (AM, 99%), *N,N*′-methylene bisacrylamide (MBAM, 99%), *N,N,N*′*,N*′-tetramethylethylenediamine (TMEDA, 99.5%), silver nitrate (AgNO_3_, >99%), *N-*(5-fluoresceinyl)maleimide (>90%), Triton X-405 (70% solution in water), cetyltrimethylammonium bromide (CTAB, 98%), ammonium hydroxide solution (reagent grade, 28–30% NH_3_ basis), tetraethyl orthosilicate (TEOS, reagent grade, 98%), (3-mercaptopropyl)trimethoxysilane (MPTMS, 95%), 3-glycidoxypropyl trimethoxysilane (GPTMS, 98%), 3-aminopropyl triethoxysilane (APTES, 98%), vinyltrimethoxysilane (VTMS, 98%), methanol, dimethyl sulfoxide (DMSO; >99.5% GC), poly(vinyl alcohol) (PVA, 80% hydrolysed, *Mw.* 10 K), poly(vinyl pyrrolidone) (PVP, *Mw.* 360 K), mercury chloride (HgCl_2_, 99%), rhodamine B (RhB, 95%), cyclohexane (99%) and light mineral oil were purchased from Sigma-Aldrich (Gillingham, UK) and used as received. Distilled water was used throughout the experiments.

### 2.2. Preparation of Polyacrylamide (PAM) and Ag/PAM Composite Monoliths

PAM beads were prepared according to the method reported previously, with the internal oil phase volume ratio of the oil-in-water (O/W) emulsion being 80 *v*/*v*% [20]. A similar procedure with varying internal oil phase volume ratios (75 *v*/*v*% and 60 *v*/*v*%) was followed to prepare PAM monoliths. Typically, a stock monomer/crosslinker solution was first prepared by dissolving 14.4 g of AM and 3.6 g of MBAM in 20 cm^3^ of 5 wt% aqueous PVA solution. To form the oil-in-water emulsion, the aqueous phase was prepared by mixing 3 cm^3^ of stock solution with 1 cm^3^ of Triton X-405 as a stabiliser and 200 µL of 10 wt% aqueous APS solution as a catalyst. The oil phase of emulsion consisted of 9 cm^3^ of light mineral oil and 60 µL of TMEDA. The emulsion was formed by adding the oil phase into the aqueous phase drop-wise and stirred at 1200 rpm for 30 min. The obtained emulsion was allowed to polymerise at 60 °C for 24 h. The internal oil phase was removed from the monolith by immersing in cyclohexane for 24 h, then washed three times with acetone and cyclohexane mixture (volume ratio 1:1) and allowed to dry in air at room temperature.

To prepare the Ag/PAM composite monolith, Ag nanoparticles were first prepared via a hydrothermal method. A stock solution of silver nitrate in water was prepared at the concentration of 5 × 10^−3^ M. Then, 0.05 g of PVP was dissolved in 5 cm^3^ of silver nitrate solution for one hour. The solution was transferred into an autoclave and the hydrothermal reaction was conducted at 180 °C for 2 h. The obtained orange Ag nanoparticle suspension was used without any further purification. AM and MBAM were dissolved in an aqueous Ag nanoparticles suspension and used for the preparation of PAM monoliths.

### 2.3. Ship-in-a-Bottle Synthesis of Silica Microspheres in Macroporous PAM Scaffolds

Both PAM beads and PAM monoliths were employed as the scaffolds. The PAM scaffolds prepared from high internal phase emulsions (>74.05 *v*/*v*% internal droplet phase), i.e., 80 *v*/*v*% and 75 *v*/*v*% in this study, exhibited highly interconnected macropores [20], while the PAM monolith prepared from the emulsion with a 60 *v*/*v*% internal phase showed relatively isolated macropores with small interconnecting windows [21]. Different silica precursors (TEOS, MPTMS, VTMS and APES) and mixing precursors (VTMS:MPTMS, APTES:MPTMS and GPTMS:MPTMS, volume ration 1:1) were used to synthesise silica microspheres with different surface functions within the macroporous scaffolds. The concentration of silica precursors in the reaction mixture was varied to adjust the size and loading of silica microspheres in the PAM scaffolds. Typically, 5 cm^3^ of aqueous solution containing organic reagents PVA (0.25 g) and CTAB (0.1 g) was prepared. To facilitate the dissolution of CTAB, 5 cm^3^ of methanol was added, followed by 2.5 cm^3^ of silica precursor (either TEOS or MPTMS). Then, the fully dehydrated PAM material (0.20 g) was added to the mixture to absorb the reagents into the macropore voids with gentle shaking on a shaker (IKA KS130 Basic) and allowed to fully saturate for 24 h. The purchased ammonium hydroxide solution (1.0 g) was diluted by mixing with 4.0 cm^3^ of water, and then 2.0 cm^3^ of the diluted ammonia solution was added into the reaction mixture. The silica particles were allowed to fully grow for 24 h at room temperature (20 °C). The composite material was filtered, rinsed with water and allowed to dry in air. The same procedure was used for both PAM beads and monoliths. The composite material was calcined in a furnace (Carbolite, CWF1200) to produce a silica material. The calcining condition was: heat at 1 °C/min in air to 550 °C, hold for 300 min and then cool down to room temperature at 10 °C/min.

### 2.4. Fluorescent MPTMS Silica Microspheres and Composite Monoliths

*N*-(5-Fluoresceinyl)maleimide (a fluorescent dye) was covalently linked to the MPTMS molecule via a thioether linker chemistry in the presence of DMSO under a nitrogen atmosphere [22]. The coupled MPTMS-fluorescent molecule was co-condensed with the nonmodified MPTMS at 0.01 M (based on the volume of water + methanol containing CTAB, PVA and silica precursor, as described above) to produce fluorescent silica microspheres. The loading of the fluorescent dye to the MPTMS silica microspheres was calculated to be 0.78 mol%, based on the typical synthesis condition, as outlined in Section 2.3. Both MPTMS silica microspheres and the composite monoliths with fluorescent properties were prepared.

### 2.5. Removal of Hg^2+^ and Rhodamine B from Water

Both a glass filter funnel with a sintered glass disc (disc diameter of 15 mm) and a stainless-steel column (4.6 mm internal diameter × 50 mm length) were used to remove organic dye RhB or Hg^2+^ from water. In the case of the glass filter, a monolith disc was formed in situ in the filter funnel. The masses of the PAM monolith, PAM–MPTMS silica monolith and packed MPTMS silica spheres in the funnel were recorded as 0.3 g, 0.35 g and 2.3 g, respectively. A volume of 200 cm^3^ of aqueous RhB solution (200 ppm) or HgCl_2_ solution (200 ppm) was passed through the filter by gravity. For the stainless-steel column, the PAM monolith (0.13 g) and PAM–MPTMs silica composite columns (0.16 g) were formed in situ in the column. The column was connected to an Agilent 1200 series HPLC (high-performance liquid chromatography) system with a quaternary pump. The aqueous RhB solution (10 ppm) was flushed through the column at a flow rate of 0.05 cm^3^/min.

### 2.6. Characterisation

Morphologies of the prepared samples were observed by a Hitachi S-4800 scanning electron microscope (SEM). The samples were adhered to the studs using Araldite resin and then coated with gold (around 15 nm thick) using a sputter coater (EMITECH K550X) for 3 min at 30 mA before imaging. Both the internal and external morphologies of the composite monoliths were examined to see the distribution of silica microspheres. Thermal stability of the composite materials was investigated by thermal gravimetric analysis (TGA, Model Q5000IR TGA, TA Instruments). The Brunauer–Emmett–Teller (BET) surface area and pore volume by N_2_ sorption at 77 K were measured using a Micromeritics ASAP 2020 adsorption analyser. Pore size distributions were calculated using the nonlocal density functional theory (NLDFT) model. Samples were degassed for 24 h at 120 °C before analysis. Macropore volumes, bulk densities and macropore size distributions were recorded using a Micromeritics Autopore IV 9500 porosimeter over a pressure range of 689–4.137 × 10^8^ Pa. Intrusion volumes were calculated by subtracting the intrusion arising from mercury interpenetration between beads or monolithic pieces (pore size greater than 150 µm) from the total intrusion. Skeletal densities were measured using a Micromeritics Helium AccuPyc 1330 pycnometer. The fluorescent emissions were measured using a UV–Vis spectrometer (Perkin Elmer, Lambda 650 S, Buckinghamshire, UK) for solid powders. For the fluorescence measurement, 0.02 g of material was used. The measurements were collected from 300 to 900 nm by using an excitation wavelength of 453 nm and scan speed of 500 nm/min. The size of particles in a suspension was measured by dynamic laser scattering (DLS) analysis on a Malvern Zetasizer Nanoseries at 25 °C from Malvern Panalytical (Malvern, UK). The concentration of RhB in water was determined by UV-Vis spectroscopy using a UV plate reader (μQuant, Bio-Tek Instruments, Harwell, UK.) with an acrylic 96-well plate. The concentration of Hg^2+^ in water was measured by inductively coupled plasma–optical emission spectrometry (ICP–OES, Agilent 5110).

## 3. Results and Discussion

### 3.1. Silica Microspheres in PAM Beads

There have been increased interests in the synthesis of functionalised organo–silica microspheres in many areas of applications [23,24,25]. The Stöber synthesis usually involved the use of TEOS as the silica precursor [25,26]. Thiol-functionalised silica precursors such as MPTMs have been increasingly used for the preparation of silica spheres because –SH groups on the silica surface provide a good platform for many potential applications, including water treatment [27,28,29].

**Figure 1:**
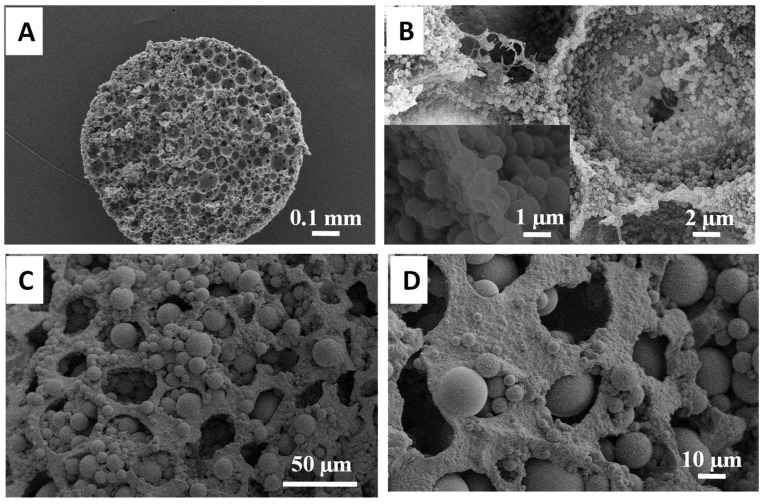
Silica microspheres were synthesised inside PAM beads using (**A**,**B**) 2.5 cm^3^ of TEOS and (**C**,**D**) 2.5 cm^3^ of MPTMS in the reaction. Cross-sectioned surface of the as-prepared (**A**,**B**) TEOS–PAM bead with the inset of B showing the silica spheres on the pore surface. (**C**) The cross-sectioned surface of the MPTMS–PAM bead. (**D**) Exterior surface of the MPTMS–PAM bead. Both (**C**,**D**) show a distribution of large silica microspheres trapped within the macropores.

In this study, both TEOS and MPTMS were used as precursors to synthesise silica microspheres within the macropores of the emulsion-templated PAM beads. The PAM beads were saturated by soaking in a solution mixture containing 2.5 cm^3^ of TEOS for 24 h before adding ammonia, to allow the reagents to uniformly diffuse into the internal pores. The hydrolysis–condensation of TEOS was induced upon the addition of ammonia and the condensation reaction was proceeded for another 24 h. This led to a mass increase of 136% for the composite beads, compared to the initial mass of PAM beads. The emulsion-templated structure was retained after this reaction (Figure 1A) [20]. A closer view of the internal pores showed a denser distribution of silica microspheres around 1 µm in diameter on the surface of the macropores, with the macropores still highly interconnected (Figure 1B and inset of Figure 1B). However, larger microspheres than the pore windows were not formed. When these composite beads were calcined at 550 °C and the organic template was removed, the silica beads generated still exhibited the emulsion-templated structure, with silica microspheres on the pore surface and the macropores’ windows completely accessible. This suggested that a coating of silica spheres was formed on the PAM surface (and penetrating into the PAM), which helped to maintain the emulsion-templated structure after calcination.

When MPTMS was used as the precursor, the resulting beads had a higher mass gain of 356%. A cross-section of the bead was examined by SEM. The bead morphology and emulsion-templated porous structure were retained (Figure 1C). Large microspheres around 5 µm in size were observed in the internal macropores. These emulsion-templated macropores were quite uniformly occupied by these large silica microspheres. The formation of large microspheres located within the external macropore surface of the beads was also observed (Figure 1D). However, the pores on the surface were not blocked and still accessible to the internal pores.

**Figure 2:**
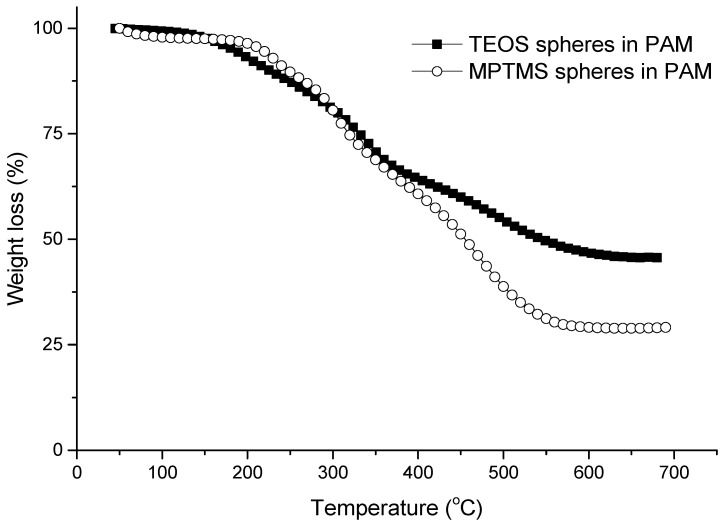
TGA profiles of silica microspheres–PAM composite beads synthesised using TEOS and MPTMS as the silica precursors, respectively.

The thermal stability and the silica content of the composite beads were further investigated. As shown in Figure 2, the mass loss was very slow until ~150 °C, which was attributed to the loss of moisture in the composites. The rate of mass loss was then increased rapidly with the temperature. This was attributed to the further loss of bound water and decomposition of organic components. A plateau of the mass loss profile was achieved at 550 °C, where the inorganic silica was fully formed. The mass loss measured by TGA indicated the presence of 28.8% silica content for the MPTMS–silica–PAM beads, while the silica content was 45.6% for the TEOS–silica–PAM beads (Figure 2). Compared to the composite beads prepared with TEOS, the additional mass loss for the MPTMS–silica–PAM beads was attributed to the mercaptopropyl chains present within the particles.

**Figure 3:**
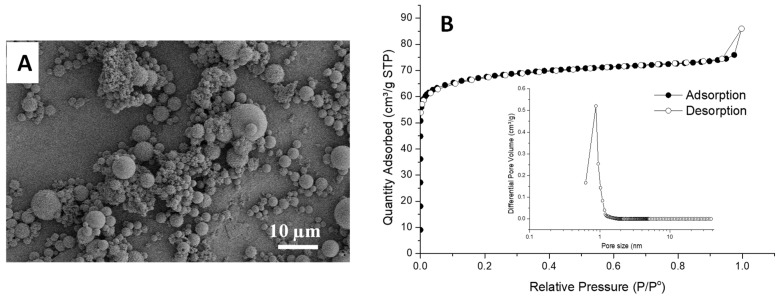
Characterisation of MPTMS–silica–PAM composite beads after being calcined at 550 °C. (**A**) The SEM image; (**B**) the N_2_ sorption profile with the inset showing pore size distribution calculated by NLDFT method.

Considering the favourable structure of MPTMS–silica spheres within PAM beads, i.e., large microspheres trapped inside the macropores with a smaller window, the MPTMS–silica composite beads were further studied by calcining at 550 °C. The bead structure was completely destroyed. SEM imaging revealed the formation of silica microspheres (Figure 3A). This indicates that no or an insufficient amount of silica was incorporated into the PAM scaffold. When characterised by N_2_ sorption analysis, the MPTMS–silica–PAM composite beads gave a low surface area around 8 m^2^/g. The silica microspheres obtained after calcining the MPTMS–silica–PAM composites beads showed a higher surface of 190 m^2^/g with micropores around 0.8 nm (Figure 3B).

**Figure 4:**
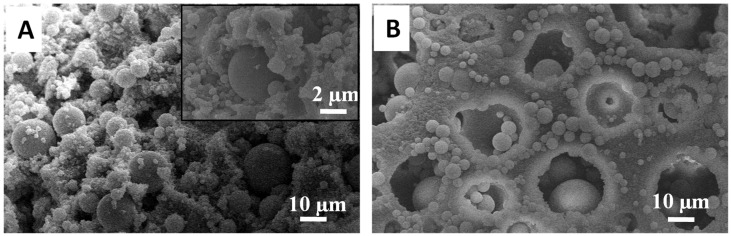
Silica microspheres were synthesised inside PAM beads by employing both TEOS and MPTMS in the reaction. (**A**) Cross-sectioned surface of the as-prepared bead with the inset showing the silica spheres on the pore surface. (**B**) Exterior surface of the as-prepared bead showing a distribution of large microspheres trapped within the macropores.

The different sizes in silica microspheres and different outcomes of calcining these composite beads suggest that the surface interactions of PAM with MPTMS and TEOS were different. This is further evidenced when the condensation of MPTMS was carried out in the presence of TEOS with the PAM beads. Figure 4 shows the structure of the resulting composite beads. The cross-sectioned surface clearly shows the presence of large microspheres with the surrounding smaller microspheres on the emulsion-templated pore wall. This indicates the sequential condensation of TEOS and MPTMS as a result of the higher reactivity of TEOS [30], instead of the simultaneous condensation of TEOS and MPTMS. At the external pore surface, both large and small microspheres (although a less dense distribution) could be observed, with the pores still accessible (Figure 4B). This may be attributed to the sol–gel process in a less-confined environment.

### 3.2. Synthesis of Organosilica Spheres in Emulsion-Templated PAM Monoliths

For some applications such as chromatography and filtration systems, a porous monolith with a defined shape can be highly advantageous [31]. Emulsion-templated PAM monoliths were thus explored for the preparation of composite monoliths. The preparation was focused on the use of MPTMS as the silica precursor because the resulting large microspheres were entrapped in the macropores, thus avoiding the possible leaching out from the scaffold, which can be highly advantageous for long-term applications. It should also be noted that the macropore voids act as miniaturised reaction vessels. Hence, the size of this void could limit the amount of reagent present during the synthesis, which would in turn affect the final particle size and distribution. In addition to this, it would be interesting to investigate the degree of influence imposed by the polyacrylamide surface on the final particle size.

**Figure 5:**
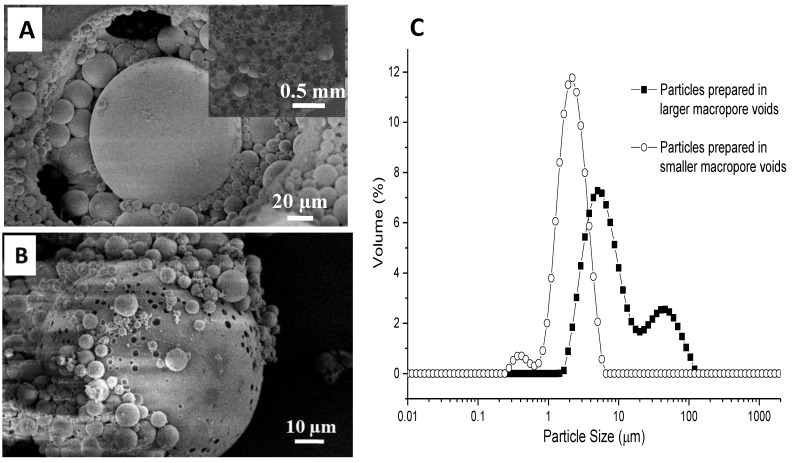
(**A**) SEM image of the composite monolith prepared with MPTMS as a precursor and the PAM monolith with large macropore voids prepared from 75 *v*/*v*% internal phase emulsion formed by stirring at 500 rpm; (**B**) SEM image of silica microspheres generated after calcining the composite in air at 550 °C; (**C**) particle size distribution of silica microspheres formed after calcination. These silica microspheres were suspended in aqueous medium and measured by DLS.

This study first investigated the effect of macropore size on the particles encapsulated within the PAM monolith. The pore size was changed by varying the oil droplet size during emulsification. As such, the PAM monolith with large macropores of 200 µm and a window size of 5 µm was prepared when forming the emulsion with a 75 *v*/*v*% internal phase by stirring at a lower rate of 500 rpm (all of the other emulsions were formed at a stirring speed of 1200 rpm). When this monolith was used as the scaffold, a broader particle size distribution of silica microspheres was obtained with particles reaching up to 100 µm in size (Figure 5A). The size distribution was measured after removing the organic components by calcination, and it showed a bimodal size distribution of around 7 and 50 µm in size (Figure 5B,C). The larger particles were formed from the MPTMS absorbed into the large pores of the PAM monolith. The smaller particles were secondary particles formed on the large silica particles when MPTMS was further diffused into the PAM monolith via the interconnecting windows during the reaction.

**Figure 6:**
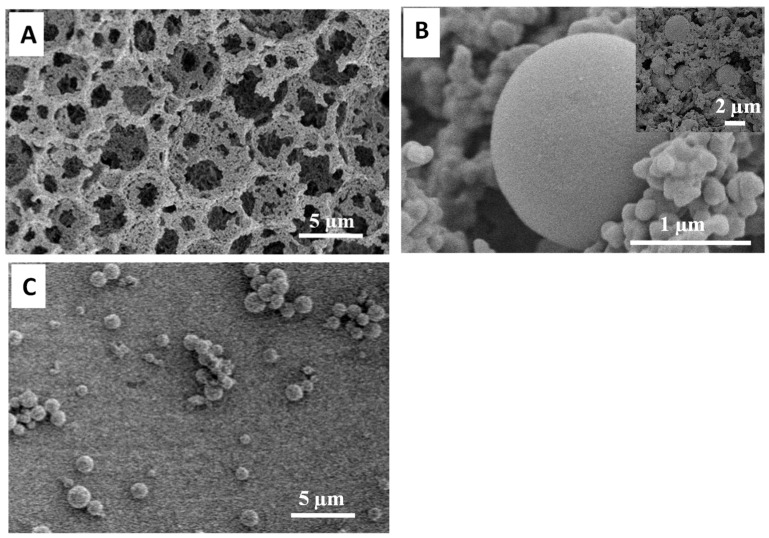
The PAM monolith with smaller macropore voids (prepared from 75 *v*/*v*% internal phase emulsion formed by homogenisation) was used for the composite synthesis. (**A**) SEM image of the PAM monolith with smaller pores; (**B**) SEM image showing silica microspheres in the composite monolith; (**C**) the silica microspheres obtained after calcination.

The PAM monolith with smaller macropore voids was prepared using the 75 *v*/*v*% O/W emulsion formed by homogenisation instead of stirring. A highly interconnected porous monolith with void sizes in the range of 3–10 µm was revealed (Figure 6A). Correspondingly, the size of silica microspheres was found to reduce to around 2 µm in the confinement of smaller macropore voids (Figure 6B). After removing the polymer scaffold by calcination, uniform silica microspheres with a narrower size distribution were observed (Figure 5C and Figure 6C). Comparing the PAM monoliths with larger and smaller voids, individual silica microspheres were encapsulated inside the macropore voids in both cases. However, the size of the silica microspheres changed corresponding to the size of the macropore voids. This was accompanied by a drop in mass gain (large voids = 290% and smaller voids = 120%), which suggested that smaller voids restricted the amount of reagents being absorbed.

**Figure 7:**
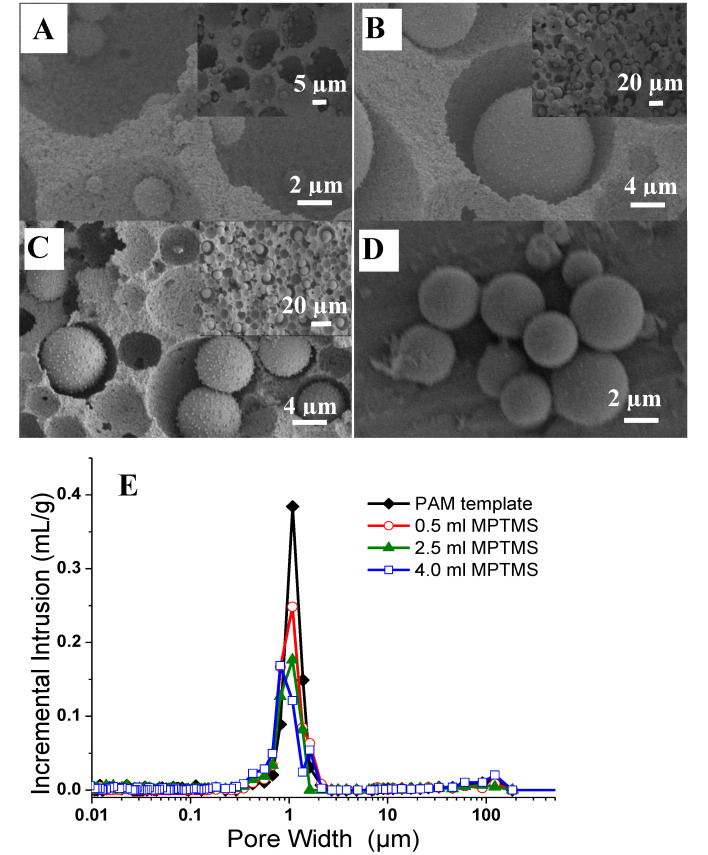
Silica microspheres were synthesised inside the PAM monolith (see Table 1, templated from 60 *v*/*v*% O/W emulsion) using (**A**) 0.5, (**B**) 2.5 and (**C**) 4.0 cm^3^ of MPTMS in the reaction. (**D**) Silica microspheres obtained after calcining the composite prepared using 2.5 cm^3^ of MPTMS. (**E**) Macropore size distribution measured by Hg intrusion porosimetry.

This system was further investigated by reducing the internal pore volume of the polymer monolith with reduced pore windows to neighbouring pores, achieved by reducing the internal phase volume ratio to 60 *v*/*v*%. This led to an increased wall thickness of the macropores and improved mechanical stability of the composites. Different amounts of MPTMS were utilised in the reaction, varying from 0.5 to 4 cm^3^ to produce a uniform distribution of silica microspheres with tuneable loading and size, while the amounts of the other reagents were kept the same. As the MPTMS content increased in the reaction mixture, this resulted in a high mass gain reaching up to 118% (Table 1 Sample S3). The emulsion-templated structure was retained after synthesis and the morphology was examined by SEM (Figure 7A–C). At 0.5 cm^3^ of MPTMS, the particles formed in the PAM macropores were around 1 µm in diameter and relatively uniform in distribution (Figure 7A; sample S1). However, silica microspheres in the range of 5–10 µm in diameter were observed by increasing the MPTMS amount to 2.5 cm^3^ and 4 cm^3^ (Figure 7B,C; samples S2 and S3 respectively). There was a denser distribution of microspheres observed in the macropores. However, it was observed that in some areas, no microspheres were present, which could possibly have fallen off during cross-sectioning the monolith because the particles were freely entrapped inside the macropore voids. As such, the size of MPTMS particles depends upon the synthesis conditions such as MPTMS concentration. The formation of large microspheres in PAM macropores at higher MPTMS concentration can reduce the possibility of the microspheres being leached out of the pores as they are too large to access the interconnecting pore windows. In all cases, the macropore surface areas were similar for the composites and were in the range of 6–9 m^2^/g (Table 1). After calcination, the templated structure collapsed and silica particles were obtained (Figure 7D).

The macroporosity of the composites was characterised by mercury intrusion porosimetry. The 60 *v*/*v*% emulsion-templated PAM exhibited an intrusion pore volume of 2.878 cm^3^/g, with a macropore size distribution around 1.08 µm corresponding to the interconnecting window between the emulsion-templated pores (Figure 7E). The intrusion pore volume decreased after introducing the silica microspheres, which suggested that some of the macropore volume was displaced by the microspheres (Table 1). As the MPTMS amount increased, the intrusion pore volume decreased to 0.945 cm^3^/g for sample S3. The macropore size measured by Hg intrusion (i.e., window size) was similar to the original PAM monolith after the growth of silica microspheres in the pores (Figure 7E), indicating that the formed silica microspheres occupied the macropore voids and the minimum number of silica spheres were formed on or around the pore windows. However, it was noticed that there was a slight decrease in pore size to around 0.81 µm at the higher MPTMS amount (sample S3). This implied that there was some sort of a partial blockage of interconnecting pore windows caused by the large microspheres.

**Figure 8:**
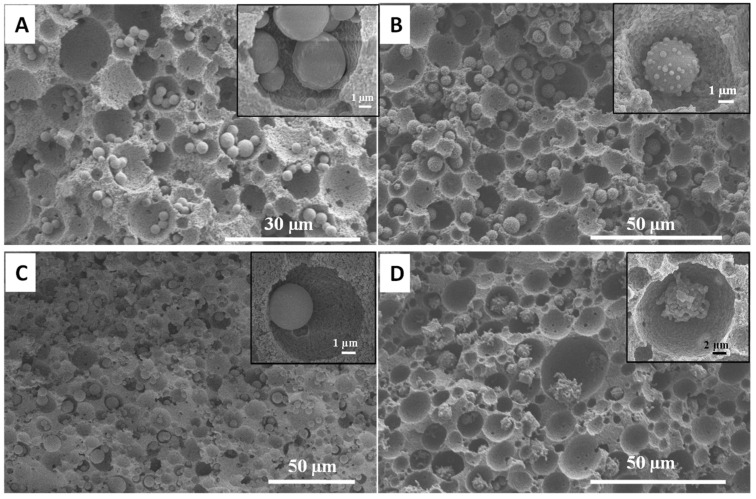
SEM images of the cross-sectioned silica–PAM monoliths prepared using different types of silica precursors: (**A**) VTMS, (**B**) VTMS:MPTMS (1:1), (**C**) APTES:MPTMS (1:1) and (**D**) GPTMS:MPTMS (1:1). The inset in each image shows the enlarged view of silica microspheres within an emulsion-templated macropore.

**Figure A1:**
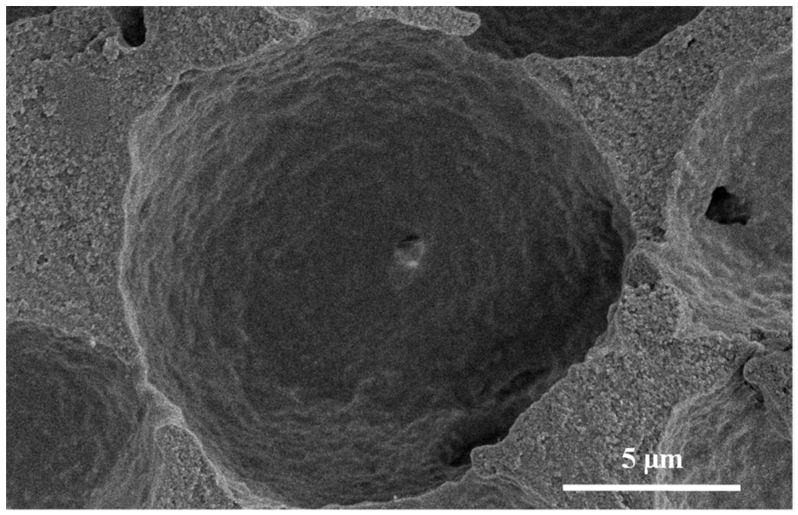
The SEM image shows the surface morphology of the silica–PAM composite monolith when APTES was used as the silica precursor.

The effect of other silica precursors was also investigated. MPTMS was replaced by or mixed with VTMS, APTES and GPTMS. The use of APTES as the sole silica precursor resulted in the surface coating of the PAM scaffold rather than spheres formation on the pore surface (Figure A1). This suggested that the polyacrylamide surface interacted with certain precursors and affected the resulting silica morphology. However, the use of VTMS as the silica precursor led to the formation of vinyl-functionalised silica microspheres in the size range of 2–5 µm within the macropores, and the silica microspheres were relatively uniformly distributed across the monolith (Figure 8A). It is believed that the co-condensation of two precursors together can strongly influence the particles’ morphology and size distribution, but provide dual functionalities [27,28,29,32,33]. Therefore, GPTMS, APTES and VTMS were mixed with MPTMS in a volume ratio of 1:1 and used as the precursors to synthesise functional silica microspheres with the macroporous monoliths. The mixture precursors promoted the formation of a uniform distribution of microspheres throughout the scaffolds (Figure 8B–D). However, the microspheres’ morphologies are different. Co-condensation of GPTMS:MPTMS and VTMS:MPTMS resulted in the formation of microspheres with a tentacle-like and nanosphere-coated surface morphology, respectively (Figure 8B,D), whereas APTMS:MPTMS as a silica precursor generated smooth-surfaced microspheres (Figure 8C).

### 3.3. Synthesis of Fluorescent Spheres in Emulsion-Templated Scaffold

**Figure A2:**
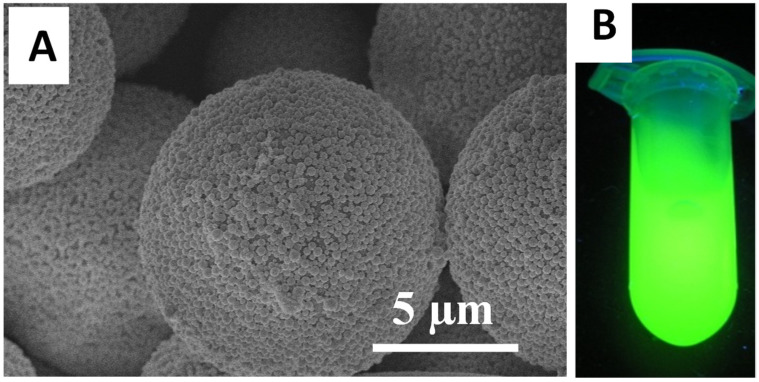
(**A**) The SEM image of fluorescent MPTMS particles and (**B**) the appearance of the MPTMS particles suspended in solution under the UV light at 365 nm.

Fluorescent microspheres/nanoparticles have been widely used for biosensing and metal ion detection [34,35,36,37]. The entrapped microspheres in a macroporous scaffold could provide a free functional surface with enhanced mass transport and easy removal [3,9,10,38]. In order to prepare fluorescent silica microspheres, *N*-(5-fluoresceinyl)maleimide was covalently linked to a MPTMS molecule via a thioether linker chemistry, where the –SH group was coupled to the maleimide group [22]. This reaction was self-driven without the need of a catalyst. The mixture was stirred overnight in the presence of DMSO under nitrogen atmosphere. The obtained fluorescent-MPTMS molecules were then used without any treatment to co-condense with the unmodified MPTMS. Throughout this study, the fluorescent agent was maintained at the concentration of 0.01 M. The obtained silica particles indicated the formation of a spheres-on-sphere morphology in the size range of 5–10 µm in diameter, as reported previously [39] (Figure A2A). The particles exhibited stronger green fluorescence under the UV light (Figure A2B), and immersing the particles in water for 24 h did not show any unbound dye molecules leached out from the surface. This indicated that the immobilised fluorescent agent was successfully co-condensed with the silica microspheres.

**Figure 9:**
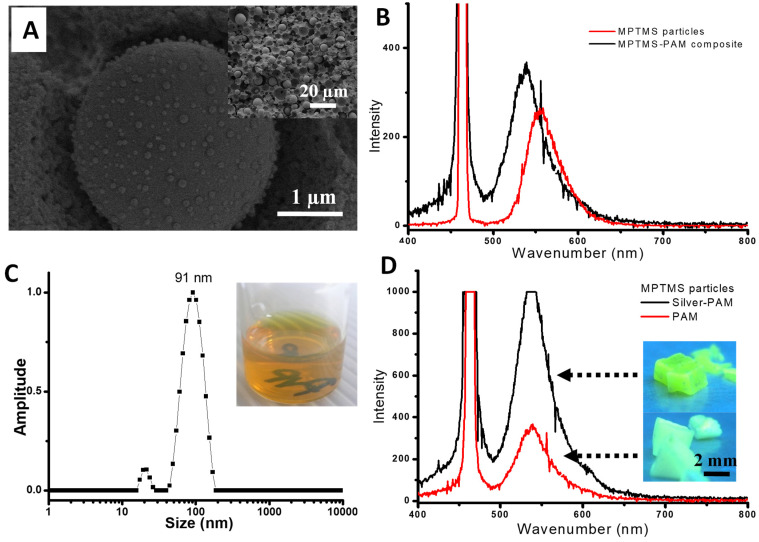
(**A**) Fluorescent silica microspheres were synthesised inside the PAM monolith with the inset showing the distribution of spheres within the scaffold. (**B**) Emission spectra of MPTMS particles and MPTMS silica–PAM composites. (**C**) DLS plot for the hydrothermally prepared silver nanoparticles stabilised with PVP 360K with the inset showing a photo of the Ag nanoparticles suspension. (**D**) Emission spectra of MPTMS microspheres synthesised inside PAM and Ag/PAM monoliths, with the inset showing the photos of the MPTMS silica–PAM and MPTMS silica–Ag/PAM monoliths under the 365 nm UV lamp.

These fluorescent microspheres were then prepared in the PAM monolith to determine the effect of polymer encapsulation on fluorescence properties. The fluorescent MPTMS microspheres were synthesised and encapsulated in the porous PAM monolith prepared with a 60 *v*/*v*% internal phase emulsion. The obtained monolith was relatively yellow in colour and showed green fluorescent emission under UV light. The internal structure was observed by SEM, which indicated that the macropores’ structure was retained after the reaction (Figure 9A). Large microspheres were formed in the macropore voids, which was similar to the original procedure (Figure 9A vs. Figure 7B). The macroporosity of the composite was characterised by Hg intrusion porosimetry, giving an intrusion pore volume of 1.075 cm^3^/g with a macropore size distribution around 1.09 µm, consistent with the interconnecting window between the emulsion-templated pores (Table 1).

Solid-state fluorescent measurement was carried out to determine the fluorescent emission intensity of the composite monolith. Figure 9B shows the fluorescent spectra of the MPTMS–PAM composite monolith. The encapsulation of silica microspheres in PAM showed enhanced emission intensity, in comparison with MPTMS silica powder. The emission maximum of the microspheres peaked at λ_max_ = 553 nm and shifted to λ_max_ = 536 nm for the composite. It should be noted that the amount of dye-modified-silica present within the PAM monolith represented approximately ~20% of the total mass of the silica powder used during fluorescence measurements. It appears that the acrylamide structure was interacting with the fluorescent molecules, which resulted in a shift in fluorescence intensity from λ_max_ of 553 nm to 536 nm. This is probably due to the alteration of the molecular orbital of the excitable electrons, e.g., changes in absorbance [40]. This resulted in a change in energy between the ground and excited states, which is known as the “Stokes shift”. The larger the difference between ground and excited state energies, as the electron decays to the ground state, the bigger the shift in emission wavelength that is expected [40].

**Figure A3:**
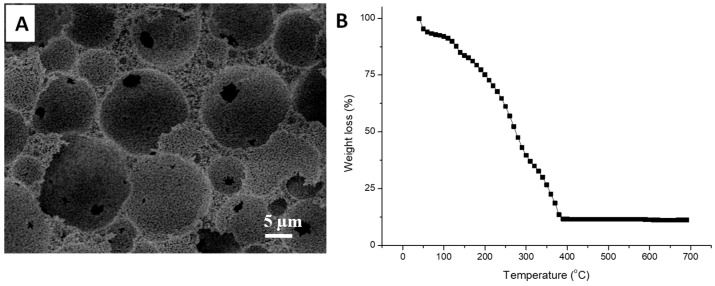
(**A**) The SEM image of silver–PAM composite with 60 *v*/*v*% internal phase volume, and (**B**) the TGA profile of the composite monolith.

Ag nanoparticles were reported to enhance the fluorescence intensity of the dye molecules and reduce their self-quenching [41,42]. In this study, the effect of Ag nanoparticles on the fluorescence intensity of the composite monolith was investigated. The Ag nanoparticles were prepared via a hydrothermal method using AgNO_3_ as a precursor and PVP as a stabiliser. The size of the nanoparticles obtained was around 91 nm and the nanoparticle suspension was very stable (Figure 9C). The Ag nanoparticles were directly mixed with the monomer (AM)/crosslinker (MBAB), which were used to prepare the porous Ag/PAM composite monolith. Ag nanoparticles were entrapped in the wall of the macropores of the PAM monolith during polymerisation (Figure A3A). The composite was analysed by TGA, which indicated the presence of 11.5% silver nanoparticles (Figure A3B). This Ag/PAM monolith was used as a macroporous scaffold to prepare fluorescent MPTMS particles. The obtained monolith showed a greater increase in emission intensity with an emission maximum of λ_max_ = 536 nm (Figure 9D). This indicated that silver entrapped in the macropore surface enhanced the fluorescence intensity. This further confirmed that the interaction between PAM and silica was occurring. This fluorescent macroporous composite monolith with enhanced fluorescent intensity may be highly useful for the sensing and detection of metal ions [37,43].

### 3.4. Monolithic Filtration System for Adsorption of Metal Ions and Organic Dye

**Figure 10:**
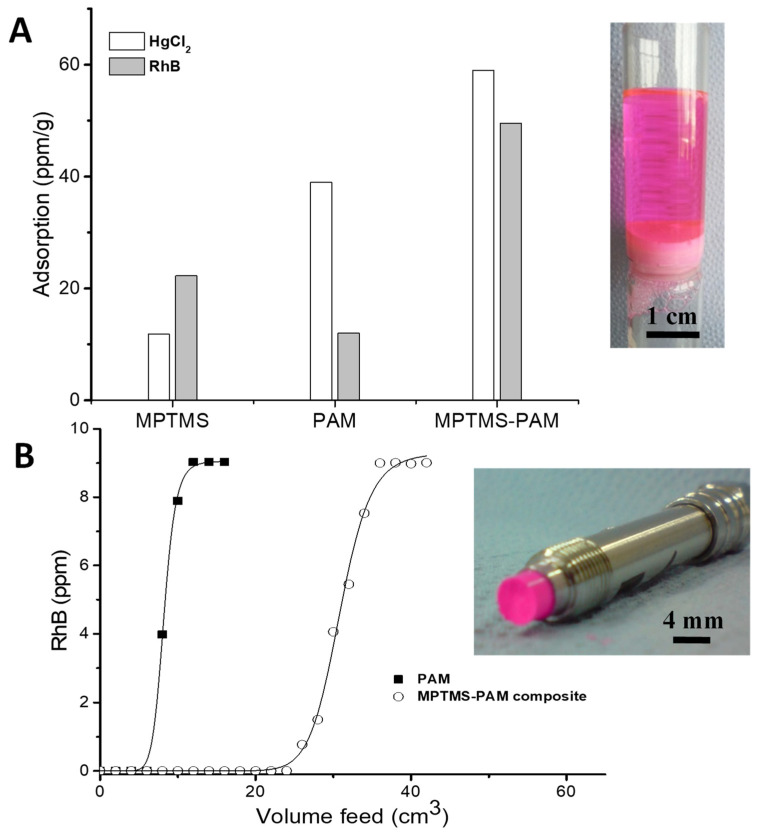
(**A**) Adsorption performance of MPTMS silica microspheres, PAM monolith and MPTMS silica–PAM composite monoliths for the removal of Hg ions (as in HgCl_2_) and rhodamine B (both concentrations at 200 ppm). The solution (20 cm^3^) was pushed through the monolith filter (as shown in the inset) by gravity. (**B**) A breakthrough flow test of RhB solution (10 ppm) with the PAM and MPTMS silica–PAM monoliths encased in the stainless-steel column. A photo of the unpacked column after the test is shown as the inset image.

PAM monoliths can be easily moulded into disk- or rod-shaped materials. The disk-shaped monolith was prepared and used as a filter for the rapid adsorption of heavy metal and organic contaminants. In this study, aqueous HgCl_2_ and RhB solutions at a concentration of 100 ppm (20 cm^3^) were used for demonstration as they have higher affinities to different functionalities, e.g., –SH groups and –NH_2_ groups [29,44]. As a control, MPTMS silica microspheres and the PAM monolith were packed separately in a sintered filter funnel. The inset of Figure 10A shows the setup of the monolithic filter for the experiment. It was found that acrylamide groups showed more interaction towards HgCl_2_ adsorption with 40 ppm/g of adsorption, whereas the –SH group favoured RhB binding with 22 ppm/g of adsorption. Encapsulating MPTMS microspheres within the PAM scaffold showed a higher adsorption of both RhB and HgCl_2_ due to the combined functionality. Thus, the adsorption of HgCl_2_ and RhB reached up to 58 ppm/g and 50 ppm/g, respectively (Figure 10A). These results demonstrate the superior performance of the composite materials for both HgCl_2_ and RhB. It should be mentioned that the results are not as high as reported in the literature, where silica nanoparticles or carbon xerogels were used for batch tests [29,44]. However, our results were obtained from the flow adsorption measurements, instead of batch tests. The monolith disk could be easily recycled from waste water with fast separation. Furthermore, both silica and PAM could be easily modified with different functional groups to target for a variety of other pollutants. This approach may be extended to other cheap and high-performance materials, e.g., alginate, carbon and biomass.

**Figure A4:**
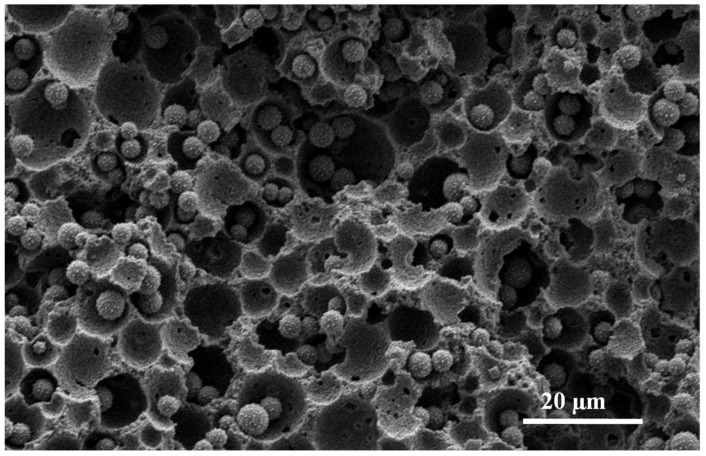
The SEM image of the cross-sectioned surface of the MPTMS silica–PAM monolith unpacked from the column used for the flow adsorption of RhB, as shown in Figure 10B.

More importantly, the composite monoliths could be fabricated in different shapes, e.g., as a column in a flow reactor for continuous flow treatments. Monolithic columns are commonly used as a preferred operation mode in most water-purification systems as they offer simplicity and rapid kinetics. In this work, the monolithic columns of PAM and MPTMS–PAM were prepared and used to demonstrate rapid separation of RhB from its water solution. The RhB aqueous solution (10 ppm) was flushed through both monolithic columns. The PAM monolith did not show high capacity for the adsorption of RhB. After the 3 cm^3^ feeding volume, RhB was detected at the outlet of the column. The composite monolith showed a significant increase in the adsorption of RhB, reaching 7× of the PAM volume capacity (Figure 10B). The structure of the monolith was examined after the adsorption study and it did not show any structural changes and deformation after unpacking the column, and the silica spheres remained encapsulated within the macropores (Figure 10B inset and Figure A4). The experimental evidence implies that this composite structure showed the ability for the effective removal of heavy metal and organic contaminants and could further be used for effective removal of other contaminants.

## 4. Conclusions

The encapsulation of large silica microspheres within emulsion-templated scaffolds was demonstrated via a ship-in-a-bottle synthesis approach. By using different silica precursors and mixing precursors, silica microspheres with multiple functional groups such as –SH, –NH_2_ and –OH groups were successfully formed in situ inside the macropore voids of the emulsion-templated PAM beads and monoliths. The size of the silica microspheres was larger than the windows interconnecting the macropores. This avoided the leaking of the microspheres from the macroporous scaffolds. The particle size of the silica could be controlled by adjusting the silica precursor concentration and varying the size of the emulsion-templated macropore voids. Fluorescent silica microspheres were prepared by co-condensing with the MPTMS modified by a fluorescent dye. The encapsulation of fluorescent MPTMS microspheres within the PAM scaffolds provided a much stronger fluorescence intensity, which was further enhanced by the incorporation of Ag nanoparticles within the polymer matrix. This fluorescent composite may provide an effective platform for the detection and monitoring of heavy metal ions. The silica–PAM composites in the form of a monolithic disk in a filtering funnel and monolithic column in a stainless-steel column were tested for the adsorption of RhB and HgCl_2_ from water. The composite showed effective removal of both RhB and Hg^2+^, indicating the high performance of combined functionalities from the acrylamide surface and –SH groups on silica microspheres. The flow test with the stainless-steel column demonstrated a more efficient removal of RhB in the composite monolith column than that of the PAM monolith column.

## Figures and Tables

**Table 1:** Characterisation data for the MPTMS–PAM monolith composites.

Samples	MPTMS (cm^3^) ^a^	Mass Gained (%)	Surface Area (m^2^/g) ^b^	Intrusion Volume (cm^3^/g) ^c^	Bulk Density (g/cm^3^) ^d^	Macropores (µm) ^e^
PAM	---	---	6.13	2.878	0.109	1.08
S1	0.5	112	9.36	1.503	0.463	1.08
S2	2.5	114	7.64	1.194	0.563	1.08
S3	4.0	118	8.69	0.945	0.595	0.81

Note: The PAM monolith was prepared from the O/W emulsion with 60 *v*/*v*% internal phase. ^a^ Standard preparation condition: 0.25 g of PVA 9K and 0.1g of CTAB, 5 cm^3^ of water, 8 cm^3^ of ethanol, 2 cm^3^ of ammonium solution and with varying amount of MPTMS (0.5, 2.5 or 4.0 cm^3^). ^b^ Measured by the BET method via N_2_ sorption. ^c^ Intrusion volumes measured by mercury porosimetry. ^d^ Measured by He pycnometer. ^e^ Calculated from mercury intrusion data.

## Data Availability

The data presented in this study are available on request from the corresponding author.

## Appendix A

More data that supplement the main text and enhance the discussion are given below.
